# Capturing Lipid Nanodisc Shape and Properties Using a Continuum Elastic Theory

**DOI:** 10.1021/acs.jctc.2c01054

**Published:** 2023-02-01

**Authors:** Itay Schachter, Daniel Harries

**Affiliations:** † Institute of Organic Chemistry and Biochemistry of the Czech Academy of Sciences, Flemingovo nám. 542/2, CZ-16000Prague 6, Czech Republic; ‡ Institute of Chemistry, the Fritz Haber Research Center, and the Harvey M. Kruger Center for Nanoscience & Nanotechnology, The Hebrew University, Jerusalem9190401, Israel

## Abstract

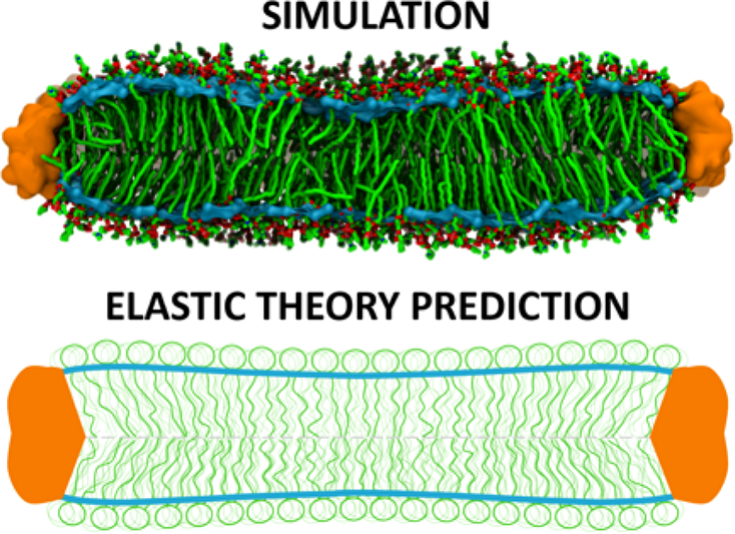

Lipid nanodiscs are nanometric bilayer patches enveloped by confining structures, commonly composed of membrane scaffolding proteins (MSPs). To resolve the interplay between MSP geometry, lipid confinement, and membrane material properties on the nanodisc shape, we apply a continuum elastic theory accounting for lipid bending, tilting, and area deformations. The equilibrium nanodisc shape is then determined by minimizing the elastic free energy functional. Analytic expressions derived under simplifying assumptions demonstrate that the nanodisc shape is sensitive to its size, lipid density, and the lipid tilt and thickness imposed at the contact with the MSP. Under matching physical parameters, these expressions quantitatively reproduce the shape of nanodiscs seen in molecular dynamics simulations, but only if lipid tilt is explicitly considered. We further demonstrate how the bending rigidity can be extracted from the membrane shape profile by fitting the numerically minimized full elastic functional to the membrane shape found in simulations. This fitting procedure faithfully informs on the bending rigidity of nanodiscs larger than ca. 5 nm in radius. The fitted profiles accurately reproduce the increase in bending modulus found using real-space fluctuation analysis of simulated nanodiscs and, for large nanodiscs, also accurately resolve its spatial variations. Our study shows how deformations in lipid patches confined in nanodiscs can be well described by a continuum elastic theory and how this fit can be used to determine local material properties from shape analysis of nanodiscs in simulations. This methodology could potentially allow direct determination of lipid properties from experiments, for example cryo-electron microscopy images of lipid nanodiscs, thereby allowing to guide the development of future nanodisc formulations with desired properties.

## Introduction

**Figure 1:**
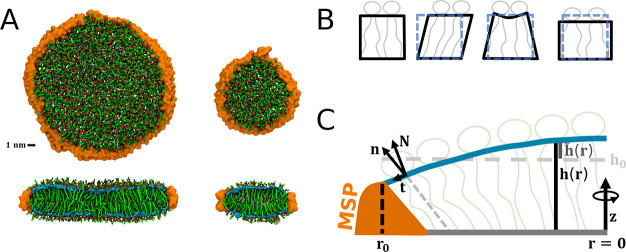
(A) Lateral (upper panel) and cross-sectional (lower panel) views of large (left) and small (right) POPC lipid nanodiscs from simulations. The scaffolding protein (MSP) is shown in orange, lipids are shown in green, and lipid phosphates are shown in red. The approximated monolayer neutral surfaces at the carbonyl position are shown in cyan. (B) Schematic of the modes of monolayer shape deformations considered by the continuum elastic theory, from left to right: undeformed, tilt, bend, area stretch. (C) Schematic cross-sectional view of a lipid nanodisc showing the vector fields described in the main text.

Lipid nanodiscs are small discoidal synthetic lipid bilayers confined by amphiphilic circumscribing structures, most typically formed of membrane scaffolding proteins (MSPs, see Figure 1A) or other polymers.^1,2^ Due to their amphipathic nature, these scaffolding structures prevent the unfavorable exposure of the nanodisc’s hydrophobic lipid core to the aqueous solution, thus stabilizing its structure.^3^ Through time, MSPs have been carefully optimized to provide a high yield of stable nanodiscs with well-defined size and lipid composition. Because of their resemblance to the native extended bilayer environment surrounding transmembrane proteins (TPs) under physiological conditions, along with their highly controlled fabrication process, nanodiscs have found numerous uses, both for TPs structural characterization and for biomedical and biotechnological applications (see e.g., ref (3)). Most notably, nanodiscs are responsible for the so-called structural revolution^4^ since they have allowed the structural determination of TPs with widely varying folds and sizes while set in the nanodisc’s native-like environment.^5−7^ Furthermore, nanodiscs have allowed insights into the role of the lipid environment in adjusting the functionality and structure of TPs.^8^

The confinement of lipids within nanodiscs impacts the lipid shape profile and local properties.^9,10^ Notably, the radial thickness profile within the nanodisc is affected by the density of confined lipids, and this strongly correlates with other local properties, such as area-per-lipid and stiffness. It is well appreciated that the lipid environment influences the relation between structure and function of TPs^11−13^ and that the lipid environment, in turn, is influenced by the TPs.^11,14^ This suggests that enveloping TPs in nanodiscs may potentially influence their structure and functionality differently than in extended bilayers of similar composition.^10^

Continuum elastic theories provide a useful framework to analyze and predict the shape and associated free energies of lipid assemblies.^15−23^ Usually, these theories are used for extended, effectively infinite lipid assemblies, described using the bending, tilt, and area compression modes of deformations, Figure 1B. Previously, our analysis of molecular dynamics (MD) simulations of nanodiscs has shown that the confinement of lipids to nanodiscs alters their elastic properties.^10^ Notably, we found up-to a 3-fold increase in the tilt and bending moduli at the center of small nanodiscs encapsuled by MSP1 (with an approximate radius of 5 nm). Our analysis further revealed a local variation in the elastic moduli between the center and perimeter of the nanodisc. However, the computational cost of these simulations limits the systematic determination of the effects of the various physical parameters on the shape (i.e., local lipid thickness and lipid director) and local material properties. Applying continuum theories to nanodiscs may, on the one hand, provide more quantitative insights into the effect of lipid confinement, while on the other hand, it can eliminate the practical hurdle of computationally prohibitive simulations.

Here, we extend and apply a continuum elastic theory to restricted bilayer patches, as in the case of nanodiscs. Specifically, the model considers lipid tilt, bend, and area compression modes for a finite surface under fixed radial boundary conditions. First, we solve the model analytically under simplifying assumptions and use this solution to evaluate the effects of the various model parameters on the shape profile of the confined bilayer. We find that increasing the nanodisc radius, while keeping average lipid density fixed, alters the thickness profile of each leaflet from concave to convex, which is in line with our previous observations in simulations. We conclude that the strong link we find between MSP geometry and the nanodisc shape should be considered when designing scaffolding structures aiming to emulate the properties of extended bilayers. Our findings further suggest that the nanodisc shape should be sensitive to the number density of lipids, which is expected to induce a significant variation in shape even in highly monodisperse nanodisc solutions fabricated using state-of-the-art methods. We find that tilt is crucially important for determining the contour shape and, even more so, for the lipid director profile. Moreover, tilting is shown to mitigate the effect of overall stiffening on the shape.

Finally, we show how the analytic theory can be used to extract the average or local bending rigidity (*K*_*C*_) of simulated nanodiscs, as long as there is a large enough spatial variation in shape and as long as nanodiscs are not too elliptically deformed. Our results are in agreement with the ones extracted using real-space fluctuation-based analysis.^10,24−27^ We suggest that this methodology should also be applicable to deriving elastic constants from lipid shapes accessible, for example, in cryo-electron microscopy images. Taken together, the continuum elastic theory presented here extends the description of extended bilayers to nanometric patches of finite and highly confined bilayers.

## Model

Consider a round, radially confined symmetric bilayer patch of radius *r*_0_, Figure 1C. The normal to the midplane for the upper monolayer is defined in the positive direction of the *z* axis, and the membrane’s neutral surface *S* is chosen as the reference surface. The local monolayer thickness is *h*(***r***) = *h*(*x*, *y*, *z*) = *z*, and the area-per-lipid (APL) is *a*(***r***), with ***r*** being the coordinates over *S*. The corresponding values for a monolayer in the undeformed extended membrane are *h*_0_ and *a*_0_. The lipid director ***n***(***r***) (pointing tails-to-head) and the monolayer local normal ***N***(***r***) define 3D unit vector fields. The free energy of each monolayer, up to second order in the deformations, can be written as^15,20,21,23^
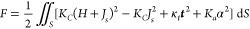
1The first two terms in the integral represent the bending energy, with the bending modulus *K*_*C*_ setting the magnitude of the energy penalty for deformations in splay, *H* = −∇·***n***, from its natural, relaxed value (defined as minus the spontaneous curvature *J*_*s*_ because curvature is defined here as positive for a spherical micelle). The third term stands for the tilt energy dictated by the tilt modulus κ_*t*_ and the tilt deformation ***t*** = ***n***/(***N***·***n***) – ***N***. The final term defines the area-compressibility energy with corresponding modulus *K*_*a*_ for deviations in the relative local APL from its planar extended bilayer value α = (*a* – *a*_0_)/*a*_0_. Considering lipid incompressibility,^28,29^ α = 1 – *h*/*h*_0_ + *h*_0_∇·***n***/2 up to first order.^23^ The nanodisc lipid–protein boundary is assumed rigid and dictates the values of *h*(*r*_0_) and ***n***(*r*_0_) at the rim. At equilibrium, the free energy functional is minimized with respect to the shape profile, described by *h*(***r***) and ***n***(***r***). For small deformation, an (*x*, *y*) parametrization of *S* is sufficient.

### Analytical Solution under Simplifying Assumptions

We first discuss the solution of the free energy minimization when small deformations are assumed, so that ***N*** ≈ −∇*h*, ***t*** ≈ ***n*** + ∇*h*, and the deformations can be described by their *xy* projections. The area differential is taken as d*S* = (1 + ∥∇*h*∥^2^)^1/2^ d*x* d*y* ≈ d*x* d*y*. Due to the divergence theorem, *J*_*s*_ is irrelevant for determining the shape and is set to 0 for simplicity. With these, eq 1 becomes
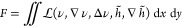
2where we have defined
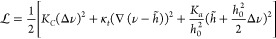
3with local excess thickness *h̃* = *h* – *h*_0_, and ***n*** = −∇ν, with ν that exists because ∇ × ***n*** = 0. The energy functional in eq 2 is minimized by solving the set of two corresponding Euler–Lagrange equations
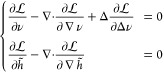
4Note that here the partial differentiation is literal so that, e.g., ∂(∇*h*)^2^/∂∇*h* = 2∇*h*. Under the assumption of spatially invariant moduli, eq 4 can be expressed as
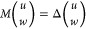
5where
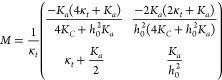
6with *u* = *h̃*, and *w* = *Δν*. Next, diagonalizing M = *P*^–1^*DP* yields the eigenvalues *q*_*i*_^2^ and the matrix elements of *P*, *p*_*ij*_. For brevity, the explicit expressions of *q*_*i*_ and *p*_*ij*_ are omitted, but these depend only on the lipid material properties and not on the details of confinement. Substituting (*ũ*, *w̃*)^*T*^ = *P*^–1^(*u*, *w*)^*T*^ into eq 5 results in
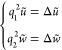
7that amount to a system of Helmholtz equations. Under radial symmetry, Δ = ∂_*rr*_ + *r*^–1^∂_*r*_ and ***n*** = *n*_*r*_***e***_*r*_ with radial unit vector ***e***_*r*_. The general solution of eq 7 is given by *ũ* = *a*_1_*I*_0_(*q*_1_*r*) and *w̃* = *a*_2_*I*_0_(*q*_2_*r*), where *I*_*k*_ is the *k*^th^ order modified Bessel function of the first kind, and *a*_1_ and *a*_2_ are complex numbers.^30^

The number of lipids within nanodiscs is assumed constant, which is true on the time scale of membrane structural relaxation.^31^ Because of lipid incompressibility, the number of lipids is then a set parameter that can be taken into account in the model by constraining the average excess thickness δ = ⟨*h̃*⟩_*S*_. This transforms the second equality in eq 4 into
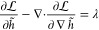
8with a Lagrange multiplier λ conjugate to δ. This has the effect of only uniformly shifting the thickness profile of the general solution up or down. Considering *P*(*ũ*, *w̃*)^*T*^ = (−∇·***n***, *h̃*)^*T*^, d(*rn*_*r*_)/d*r* = *r*∇·***n*** allows the determination of the shape profile
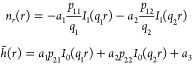
9The real number *a*_3_ stems from the thickness constraint. This solution was previously derived using a less detailed route by Akimov et al.^23^ who focused on membrane pore structure in an extended bilayer rather than the confined bilayers considered here.

The shape profile for specific boundary conditions is found by solving
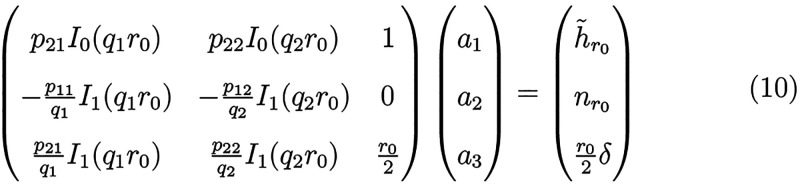
10Therefore, the *a*_*i*_’s depend on both the physical properties and confinement details. In practice, eq 9 and eq 10 were solved numerically using a code implemented in Python^32^ and Scipy.^33^

## Influence of Physical Parameters on Membrane Shape

In this section, we assess the individual influence of the model parameters on the lipid shape profile as determined by the analytical solutions derived in the previous section. To isolate the impact of each parameter, we vary them in turn, while keeping all others fixed.

### Nanodisc Radius

**Figure 2:**
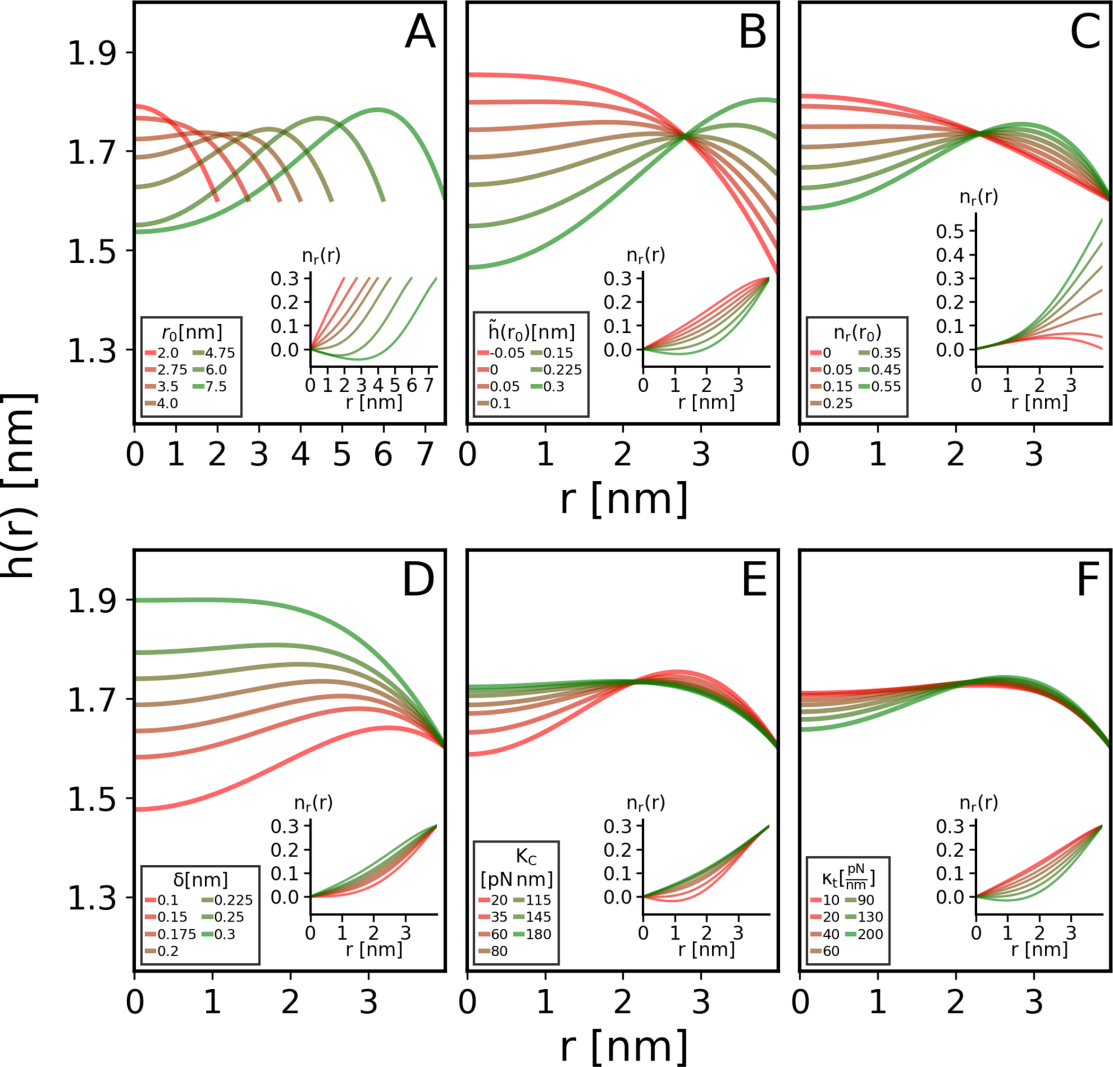
Influence of physical parameters on the nanodisc lipid shape profile determined by the analytical solution. Each panel shows the influence of a single parameter on the radial lipid height (main panel) and lipid director projection (inset) profiles. Variations are considered in (A) nanodisc outer radius, *r*_0_, (B) hydrophobic mismatch with the MSP described by *h̃*(*r*_0_), (C) lipid orientation at the rim, *n*_*r*_(*r*_0_), (D) average excess thickness, δ, corresponding to lipid number density in a nanodisc, (E) bending modulus, *K*_*C*_, and (F) tilt modulus, κ_*t*_. Each variation is made around the following physical parameters, typical for lipids in a nanodisc as found in our simulations (see Table S2): *K*_*C*_ = 80 pN nm, κ_*t*_ = 60 pN nm^–1^, *K*_*a*_ = 120 pN nm^–1^, *r*_0_ = 4 nm, *n*_*r*_(*r*_0_) = 0.3, *h̃*(*r*_0_) = 0.1 nm, and δ = 0.2 nm.

Changing *r*_0_ from small to large values results in a concave-to-convex thickness profile transition that is most prominent in the center of the nanodisc, Figure 2A. The transition agrees with our previous findings from simulations:^10^ smaller nanodiscs show a thickening in the center while larger ones have a dip. Because *n*_*r*_(*r*_0_) > 0 (implying tilted lipids at and toward the rim), the monolayer always thickens going from the boundary toward the center. By symmetry, ***n*** and ***N*** ∼ −∇*h* must approach zero at the nanodisc center, which requires membrane deformations along the radial direction. Deformations near the rim are less energetically favorable compared to those at the center because more lipids reside closer to the boundary due to the radial dependence of the annuli’s areas. Indeed, we find that larger nanodiscs are less curved near the boundary and more strongly deformed at the center. Specifically, to compensate for the increased thickness close to the rim, the thickness in the center *h̃*(0) is less than the average excess thickness δ. This trend was also found in simulation of a large nanodisc composed of POPC encapsuled by MSP2N2 protein, with an approximate radius of 9 nm,^10^ as well as in planar extended bilayers at some distance from perturbing TPs, in both theory and simulation.^34−37^ By contrast, for small nanodiscs that are highly confined, near-rim deformations are unavoidable. The radius at which the concave-to-convex transition occurs, *r*_*tr*_, depends on the choice of physical parameters, Figure S1, and is further discussed in the following sections.

We note that the analytical *n*_*r*_ profile can become locally concave near *r*_0_ (Figure 2, insets) which we have not found in corresponding simulations. This could be due to a certain extent of nanodisc ellipticity observed in simulations, which is not accounted for in the analytical model. Taken together, the elastic model not only verifies the link we have previously found between confinement radius and nanodisc shape^10^ but also provides a simple expression for the thickening close to the boundary versus the nanodisc center.

### Lipid–Protein Contact at the Rim

Figure 2B demonstrates how the nanodisc shape is impacted by the interaction of lipids at the interface with the circumscribing protein, MSP. This boundary condition is described by the deviation in monolayer thickness at the rim, *h̃*(*r*_0_), compared with the unperturbed value as imposed by the protein, i.e. the hydrophobic mismatch at the lipid–protein contact. Figure 2C describes the changes in shape when the lipid tilt angle described by *n*_*r*_(*r*_0_) at the protein–lipid interface is varied.

Analogously to increasing the nanodisc radius, increasing *h̃*(*r*_0_) at a constant radius induces a concave-to-convex thickness profile transition, Figure 2B. The number of lipids within a nanodisc, represented by the constraint on the average excess thickness, plays a key role in this profile transition (see Figure S1C), because thickening near the rim necessarily dictates thinning at the center. Interestingly, Figure 2B shows an isopachous point where all curves cross, indicating that the shape is a linear combination of 2 different asymptotic solutions of eq 10. This is also a direct result of the average thickness constraint, eq 8.

To reduce the penalty for tilting, the lipid director ***n*** tends to follow the surface normal ***N*** ∼ −∇*h* ∼ −(d*h*/d*r*)***e***_*r*_. Thus, the hydrophobic mismatch *h̃*(*r*_0_) similarly affects d*h*/d*r*(*r*) and the radial projection of the lipid director *n*_*r*_(*r*), Figure 2B, inset. The hydrophobic mismatch is also related to lipid identity, because the chain length correlates with membrane thickness *h*_0_. But changing the lipid length or *h*_0_ while keeping the hydrophobic mismatch *h̃*(*r*_0_) constant only shifts the thickness profile up or down, Figure S2A. Therefore, resulting deformations more sensitively depend on the extent of hydrophobic mismatch than on lipid identity (chain length) or MSP thickness alone. This finding agrees with simulations^10^ where we have found that the most concave medium-sized nanodiscs were composed of DLPC, the shortest chained lipid that is also expected to have the highest (positive) hydrophobic mismatch.

Increasing *n*_*r*_(*r*_0_), Figure 2C, impacts the thickness profile similarly to increasing *h̃*(*r*_0_). This stems from the thickening close to the boundary because the lipids at the rim stretch as they tilt. Here, we considered *n*_*r*_(*r*_0_) > 0 (lipids tilting toward the MSP), which is also the case in simulations; solutions for *n*_*r*_(*r*_0_) < 0 are shown in Figure S2B.

Taken together, we conclude that the nanodisc shape profile and local properties are strongly influenced by the scaffolding protein. The nanodisc shape is relevant because it correlates closely with local material properties, such as the elastic moduli.^10^ Importantly, the conformational state (and hence the function) of TPs confined within nanodiscs can be modified by the properties of the lipid environment. Because the orientation of the lipid director near the rim, *n*_*r*_(*r*_0_), and the corresponding thickness, *h*(*r*_0_), are related to the MSP structure at the interface with the lipids, controlling the MSP shape and properties could provide a way to modify the conformational state and function of proteins embedded in the nanodisc.

### Lipid Number Density

Figure 2D shows how the difference between lipid number density in the nanodisc and the planar extended (unperturbed) bilayer lipid density, represented in the model by the average excess thickness compared with the planar extended bilayer, δ, impacts the nanodisc shape profile. The lipid density, in turn, is determined by the number of lipids confined to the nanodisc and its size. We discuss positive values of δ (corresponding to smaller average area-per-lipid in the nanodisc than in the planar extended bilayer), as only these were observed in the simulations; results for negative values of δ are in the SI, Figure S2C. We find that thickness *h*(*r*) increases with δ with a concomitant increase in *n*_*r*_(*r*) due to the close correspondence between ***n***(r) and ***N***(r) ∼ −∇*h*.

The number density of lipids in a nanodisc is relevant for at least three reasons. First, membrane thickening, expected for a higher number of lipids (and hence density) in the nanodisc,^9^ overall increases the elastic moduli and thus changes the local lipid environment.^10^ Because different experimental fabrication methods of lipid nanodiscs yield different average lipid number density,^38^ this should result in distinct shape profiles. Second, the sensitivity of the shape profile to lipid number density is tightly linked to the expected polydispersity in density within a specific preparation of nanodiscs. For some fabrication methods, a high degree of monodispersity in density has been measured, with a standard deviation of ca. 3% in the number of lipids.^3^ This ±3% deviation roughly corresponds to δ_±_ = 0.20 ± 0.05 nm. Thickness profiles for δ = δ_+_ and δ = δ_–_, both included in Figure 2D, are different by ≈13% in membrane thickness at *r* = 0. This demonstrates that even small polydispersity is expected to result in significant thickness variations in a single preparation. Finally, nanodiscs with the same MSP perimeter but fewer lipids (hence smaller surface density and larger average area per headgroup) tend to deform elliptically.^38^ This should slightly mitigate the effect of lipid number density on the shape profile that is predicted by the model, because elliptical deformations can reduce the total area of confinement, thus alleviating overall thinning of the confined bilayer. Therefore, for realistic nanodiscs, δ is expected to depend sublinearly on the lipid number density for densities lower than the planar extended bilayer lipid density but linearly for higher lipid number densities, where no bilayer thinning is required and thus elliptical deformations may be neglected. In our nanodiscs simulations, δ > 0, so that elliptical deformation may be neglected for small variations in the lipid number density. Experimentally,^38^ even for nanodiscs with a lower number of lipids, the correlation between lipid number density and ellipticity is not statistically significant for ±5% variations in the lipid number density. We conclude that our model should be accurate for most current realistic nanodisc preparations, and for existing low density preparations, the model should only slightly exaggerate the effect of the lipid number density on the nanodisc shape profile. To fully address the effect of nanodisc ellipticity, future extensions of the current model should include this additional degree of freedom.

### Elastic Moduli

Increasing *K*_*C*_ or decreasing κ_*t*_ results in a convex-to-concave thickness profile transition, Figure 2E-F. This effect originates from the interplay between tilt and splay energies,^21,22,34^ which can be understood as follows. Increasing *K*_*C*_ makes splaying unfavorable, which leads to less variation in ***N*** along the radial direction. Because ***N***(*r* = 0) = 0, ***N***(*r*_0_) ∼ −d*h*/d*r*(*r*_0_) decreases at the expense of increasing the tilt. This in turn thins the lipid monolayer near the rim and thickens it near the nanodisc center. Conversely, increasing κ_*t*_ makes tilting unfavorable, thus ***N***(*r*_0_) increases so as to conform to ***n***(*r*_0_). As a consequence, |d*h*/d*r*(*r*_0_)| increases, resulting in monolayer thickening at the rim and its thinning at the center.

The nanodisc shape is more sensitive to variations in *K*_*C*_ than to variations in κ_*t*_. Although the effect of κ_*t*_ is secondary to *K*_*C*_, stiffening of the nanodisc due to lipid confinement increases both *K*_*C*_ and κ_*t*_, as the two are strongly correlated.^10^ The range of *K*_*C*_ = 20 to 180 pN nm considered here corresponds to the one we found in simulated nanodiscs.^10^ We also include results for κ_*t*_ ranging from 10 to 200 pN nm^–1^ which brackets the entire observed range of local κ_*t*_ in our simulations, which was 30 to 180 pN nm^–1^. This range of tilt modulus was selected in order to demonstrate that varying *K*_*C*_ impacts the shape more than varying κ_*t*_. Due to the opposing effect of elevating *K*_*C*_ and κ_*t*_ on their own, overall stiffening of a realistic membrane is expected to more closely follow the impact of increasing *K*_*C*_ alone, with some mitigation due to the concomitant rise in κ_*t*_, as shown in Figure S2D-F.

The necessary thickening near the rim can relax over some length scale that depends on the system parameters. However, if the nanodisc is smaller than this required length scale, a concave shape profile develops. The transition radius, *r*_*tr*_, expectedly grows with *K*_*C*_ and decreases with κ_*t*_ (Figure S1). The transition radius between concave and convex profiles at the center is linked to the typical wavelength of oscillations near deformations. This wavelength is  for planar extended bilayers,^22^ which qualitatively agrees with our findings that *l* scales with the elastic moduli similarly to *r*_*tr*_. And yet, we find that altering the thickness (Figure S1A) or tilt (Figure S1B) at the rim, as well as the constrained average thickness (Figure S1C), impacts *r*_*tr*_ more than changing the elastic moduli (Figure S1). This highlights the greater sensitivity of the shape in nanodiscs to the details of confinement.

The necessary correlation between the different parameters, such as the bending and tilt moduli, which is found in simulations,^10^ is unaccounted for in the analytical solution. However, as we show in the next section, the deformations and deviations from the flat configuration found in simulated nanodiscs are in quantitative agreement with the analytic model. Thus, the discussion of the analytic model provides a tractable yet accurate account of the impact of the various parameters on the nanodisc shape.

## Linking with Simulations

To test the applicability of the elastic model to lipid nanodiscs, in this section, we show how profiles derived from the simulations compare with the shape derived from the analytical solution under appropriate physical parameters. First, we describe the procedure for extracting from MD simulations the parameters used as model parameters and the corresponding nanodisc shape profile. Next, we compare the shape profiles observed in simulations to those predicted by the model. Finally, we demonstrate how model fits to the shape profile in simulation can directly report on the bending rigidity.

### Simulations Analysis

The majority of the simulations used for comparison has been previously described.^10^ Details of protocols applied to additional simulation used for this study are in the SI section S2 and Table S1. Elastic moduli and the radial profiles of both the bending modulus *K*_*C*_ and the MSP density are extracted following the scheme described in ref (10). Bulk area compressibility modulus, *K*_*a*_, for planar extended bilayers, estimated from using simulations of periodic bilayers, is evaluated from the area fluctuations^29^ using *K*_*a*_ = *k*_B_*TA*_0_/(2*Var*(*A*)) , where *A* is the monolayer instantaneous lateral area and reported in Table S2. Standard errors are estimated by the 10-chunk block-averaging method.^39^ The shape profiles from simulations, described by continuous functions of *h* and *n*_*r*_, were obtained using the procedure described in the SI section S3.

### Nanodisc Shape from Analytic Solution Well Matches Simulations

Analytical solutions were derived for different nanodiscs using physical parameters chosen as follows. The nanodisc radius in the analytic solution, *r*_0_, was set to the nanodisc radius as defined in simulations, *r*_*max*_ (for values of *r*_*max*_ see Table S3). The thickness and lipid director at the rim, as well as the average excess thickness δ (up to *r*_0_), were matched with their values in the corresponding shape profile in the simulation. Radially averaged values of the elastic moduli up to *r*_0_ were used in the analytic calculation. The bulk values of area compressibility modulus *K*_*a*_ and thickness *h*_0_ were extracted from unconstrained periodic bilayers simulations and used in the analytic nanodisc calculations. To derive the limit where tilt is prohibited, a value of κ_*t*_ → ∞ was used.

**Figure 3:**
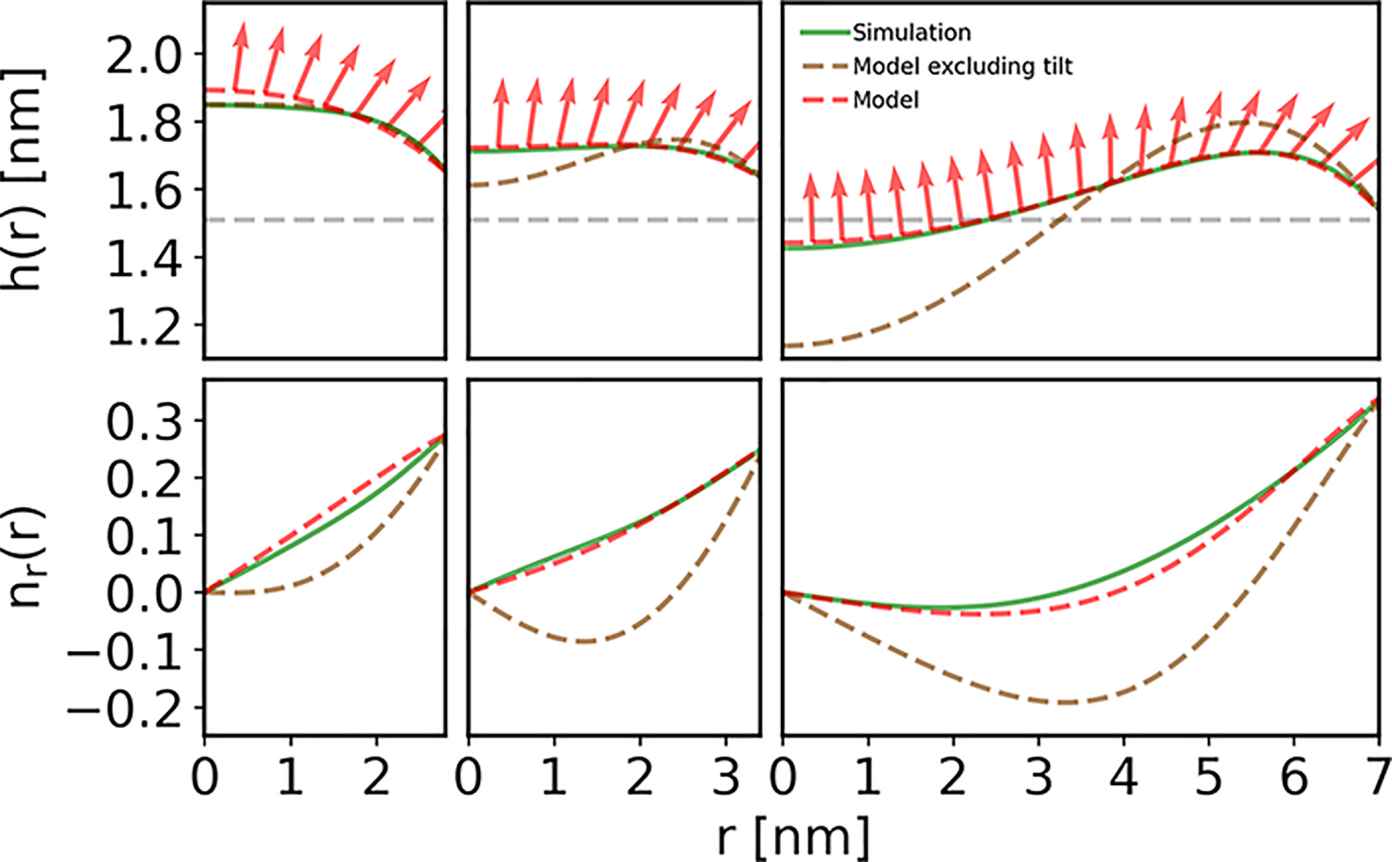
Comparison of nanodisc shape profiles found in simulation and calculated from the continuum theory. The shape profile is described by the thickness (upper panels) and radial projection of the lipid director normal (lower panels) radial profiles. Shape profiles for POPC lipid nanodiscs are shown for small (left column), medium (middle column), and large (right column) nanodiscs. Profiles found through simulation (solid line) are compared with the analytical solutions (dashed lines) under matching model parameters, as detailed in the main text. Calculations are shown with or without including the tilt degree of freedom. The red arrows illustrate lipid director orientation in calculations which allow tilt, and these correspond to the dashed red curves in the lower panels. POPC bulk thickness in simulations is shown for reference as a dashed gray horizontal line.

We compare the simulated and model profiles for nanodiscs composed of POPC, Figure 3; nanodiscs with other lipid compositions are shown in Figures S3–S7. Throughout the discussion, we define small, medium, and large nanodiscs as ones encapsuled by MSP1, MSP1E3D1, and MSP2N2 proteins, corresponding to approximate radii 5, 6.5, and 9 nm.

For small nanodiscs, the analytic model yielded a more concave thickness profile. Because in simulations the center is stiffer than the rim while in the analytic calculation only one value is used, the simulated thickness profile relaxes faster near the rim through high bending, which also lowers the thickness at the center compared to the model. Yet overall, the continuum theory adequately explains the observed deformations within simulations of small nanodiscs. Continuum theories are expected to fail when lipid patches are small, but we find that our model faithfully reproduces profiles for rather small nanodiscs that contain as few as 85 lipids per monolayer.

The match between simulations and model is even more striking for medium and large nanodiscs where it is almost entirely predictive. For these systems, we find a root of mean squared error (RMSE) of less than 0.03 nm, except for the large POPE–POPG nanodiscs with an RMSE of 0.05 nm. The model’s success in describing these nanodiscs is due to the larger system size (around 130 and 350 lipids for the medium and large nanodiscs) and to the more adequate assumption in the analytical model of spatially invariant elastic moduli, which simulations show is more appropriate for larger nanodiscs.^10^ The modest deviations of the model from the observed profile for the large POPE–POPG nanodisc may be due to lipid sorting within it (Figure S8), which is unaccounted for by the model. This lipid sorting should impact the local material properties of the lipid,^40^ as well as the resulting shape profile.

#### Importance of Tilt

Comparing the shape profiles derived from the model, with versus without the inclusion of tilt (Figures 3 and S3–S7), demonstrates that including the tilt degree of freedom yields a more concave thickness profile and smaller variations in the lipid director profile, that generally much better correspond to the simulated profile. Arresting tilt requires the monolayer’s surface to bend more, whereas allowing tilt increases the thickness at the center by reducing the thickness profile slope |d*h*/d*r*| near the boundary. For all but the small DEPC, DLPC, and POPC nanodiscs, the RMSE of the thickness profile with allowed tilt is at least half of that when tilt is prohibited. For the small nanodiscs, the thickness profiles are slightly better matched when omitting tilt, at the expense of high deviations in the lipid director profiles, with at least a 3-fold increase in RMSEs. We conclude that tilting is critical for correctly describing nanodisc shape profiles.

Tables S2 and S3 show that, on average, lipid confinement increases the average bending rigidity of most bilayers by roughly 40% and as much as ≈120% for DPPC nanodiscs. This stiffening stems from the positive average excess thickness (smaller APL) observed in the simulations compared with the corresponding planar extended bilayers, δ ≈ 0.2 > 0. The average increase in *K*_*C*_ compared to its bulk value is substantially smaller than the local variations seen in stiffness within each nanodisc in the simulations, which can be up to a factor of 3. We return to discuss this point in the next section. Surprisingly, the nanodisc size does not strongly correlate with the extent of stiffening, although small nanodiscs tend to be slightly stiffer (with the exception of DEPC).

### Resolving Bending Rigidity through Nanodisc Shape Analysis

In this section, we describe a methodology to extract the full *K*_*C*_ profile (or its average value) from fits to the shape profile found in simulation. We then further validate this new methodology by comparison to the known values of the bending rigidity in simulations. The method works best where the equilibrium shape of the lipid patch is not flat, so that significant variations in shape can be fit. We compare the findings from this methodology with real space fluctuations analysis,^25^ which is currently the only method that is able to resolve locally varying bending rigidity in finite lipid patches. The thickness profile of lipid nanodiscs has been shown to be accessible through cryo-EM.^41^ Additional model parameters should be experimentally accessible, including the lipid density determined by mass spectroscopy^3,38^ and the tilt modulus from scattering experiments of extended membranes^42^ or, as demonstrated here, from simulations. Thus, we suggest that the new methodology presented here could potentially be applied to the extraction of the bending rigidity from experimentally determined nanodisc shape profiles.

To directly derive the spatially varying bending elastic modulus from the simulated nanodiscs’ shapes, we fit the nanodisc shape predicted by the theory to the simulated one by optimizing the bending rigidity parameter. The methodology, referred to as shape analysis, is fully detailed in the SI section S4. Nanodiscs with larger *r*_*max*_ lend themselves better to this methodology, because they allow the deformations resulting from the boundary conditions to evolve more along the nanodisc radius, making the shape more sensitive to the value of the elastic moduli. This is analogous to the well-known improvement in determining surface tension with the pendant drop method when using larger drops.^43,44^ Another parameter that should be carefully chosen is the value of the boundary radius in the model, *r*_0_: on the one hand, decreasing *r*_0_ reduces the amount of information about the shape profile, while on the other hand, increasing *r*_0_ includes information about the shape far from the center, which tends to deviate from the model due to the nanodisc ellipticity. With these considerations, we chose a value of *r*_0_ < *r*_*max*_ so as to optimize information on the profile and minimize errors due to ellipticity. Taken together, the bending rigidity can be successfully extracted only for discs with *r*_*max*_ larger than ≈5 nm. For large nanodiscs, the methodology is even sensitive to spatial variations of the elastic modulus. For these nanodiscs, *K*_*C*_ is assumed to locally depend on thickness. Then, the predicted *K*_*C*_ profile up to *r*_*max*_ is determined from the fitted thickness-dependence of the bending modulus applied to the simulated thickness profile. The spontaneous curvature *J*_*s*_ is neglected for all but the large POPE–POPG nanodisc, as only POPE has a spontaneous radius of curvature comparable to the membrane thickness; the profile obtained for the large POPE–POPG nanodisc when neglecting *J*_*S*_ is in Figure S10.

**Figure 4:**
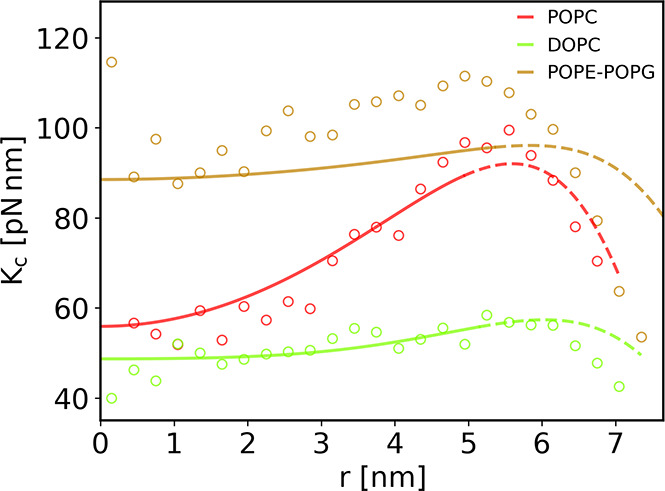
Bending rigidity radial profiles of large nanodiscs. The profiles were extracted from simulations using two different methods: real space fluctuations analysis (scatter) and through shape analysis performed up to a radius *r*_0_ (solid lines) and extrapolated up to the radius of lipid-MSP contact *r*_*max*_ (dashed lines), as described in the main text.

Figure 4 shows the radial bending rigidity profile recovered for large nanodiscs. The *K*_*C*_ profiles extracted using our shape analysis methodology are in good agreement with the ones derived using real space fluctuations analysis.^10,25^ This validates both methods for obtaining *K*_*C*_ in the nanodisc. Although, as we have shown, the influence of κ_*t*_ on the shape is secondary to that of *K*_*C*_, we find that using the value for κ_*t*_ evaluated from fluctuation analysis^25^ in the fitting procedure allows a good fit with the bending modulus derived by the shape analysis. We note, however, that for shape analysis the reference surface should be carefully chosen, because the theory requires that the reference and neutral surfaces coincide. Here, shape analysis performed well for the chosen surface located at the carbonyls, which is close to the pivotal plane^45−47^ (i.e., the surface of constant area under bending). This result is expected, at least for DOPC and POPC, for which the pivotal plane and the neutral surface coincide due to the low spontaneous curvature.^45^ Setting a deeper reference surface, defined at the third carbons of the lipid chains, led to a significantly worse fit for DOPC and POPC, see Figure S9. The sensitivity we find of shape analysis to the choice of reference surface should be carefully considered when applying this methodology to data from simulations or experiments. Nevertheless, the profiles found using both methodologies agree best for POPC and worst for POPE–POPG. An improper choice of reference surface, the small variability of *K*_*C*_ and the lipid sorting found in simulations (Figure S8) but unaccounted by the model, may make it harder to recover *K*_*C*_ from the POPE–POPG nanodisc shape. Because *K*_*C*_ was locally evaluated from the shape solely by assuming its dependence on the local thickness *h*, we can conclude that it fully correlates with bending rigidity in the nanodisc, as previously noted.^10^ The link between local membrane thickness and rigidity demonstrated using the two methodologies (shape and real-space fluctuations analyses) should not be too surprising. Specifically, thicker membranes with smaller area per lipid have been shown to be stiffer.^48,49^ Our results suggest that this thickness-stiffness relation is also relevant to local variations.

For medium-sized nanodiscs only the average bending rigidity can be faithfully extracted, Table 1. Fitted values *K*_*C*_^*fit*^ deviate from the averaged ones in simulations  by less than 3% for DPPC and DEPC and by 25% for DOPC. For other medium-sized nanodiscs containing POPC or DLPC, the bending rigidity could not be accurately determined by the fit. The methodology may fail for these nanodiscs because they are more elliptical than the rest.^10^ The optimized choice of *r*_0_ = 1.9 nm yielded a relatively good fit of *K*_*C*_ for most of the medium-sized nanodiscs. Table 1 demonstrates that circular medium-sized nanodiscs deform as expected from the continuum elastic theory and even allow extraction of an apparent average *K*_*C*_ from the shape profile.

**Table 1:** Bending Rigidity of Medium-Sized Nanodiscs

composition	[pN nm]^a^	*K*_*C*_^*fit*^[pN nm]^b^
DEPC	84 ± 1	86
DOPC	59.7 ± 0.6	44
POPC	95 ± 1	189
DPPC	147 ± 2	150
DLPC	84 ± 1	39

a Derived using real-space fluctuations analysis.^10,25^

b Derived using shape analysis as detailed in the main text and SI section S4.

## Conclusions

Continuum elastic theory captures the shape variations of lipid patches confined within nanodiscs. Supporting the applicability of the theory to lipid nanodiscs, we derived an analytical expression for the shape profile under simplifying assumptions, which matched those observed in corresponding molecular dynamics simulations. Varying model parameters and analyzing the resulting shape profiles have allowed us to demonstrate their individual impact. The shape profile is highly controlled by the nanodisc size, hydrophobic mismatch, and tilt at the rim, which are all set by the MSP. We then demonstrated how the local or average bending rigidity within nanodiscs can be resolved using a modified continuum theory that is fitted to the shape profile derived from simulations. We propose that an analogous method can be implemented for obtaining the bending rigidity from experimentally determined shape profiles of lipid nanodiscs through, e.g., cryo-electron microscopy imaging.

Our methodology highlights that bending rigidity and thickness are mutually and locally dependent. Importantly, we find significant spatial variations in the elastic moduli on the subnanometric scale that may significantly affect the energetics of biologically relevant membrane remodeling processes such as fusion or budding. We find that although bend is the most important elastic degree of freedom from the perspective of energetic relaxation, considering the lipids ability to tilt is necessary for correctly capturing the nanodiscs’ shape profile. Specifically, lipid confinement generally stiffens membranes, whereas tilt mitigates to some extent the stiffening effect on the shape profile. Our model further predicts strong variations in nanodisc shapes even in considerably monodisperse samples or between different fabrication methods. All of these unique properties of nanodiscs, that are absent from extended lipid bilayers, should be carefully considered when studying the structure–function relation of TPs embedded within nanodiscs. We suggest that carefully engineering nanodisc composition and details of confinement should allow significant modulations of the shape profile and material properties. For example, a relevant pharmacological application could involve the design of responsive MSPs that change their geometry under different solution conditions and thus alter the confined bilayer properties.

## Supporting Information Available

Details of methods for extraction of simulated shape profiles and shape analysis methodology, table with details on simulations, tables with structural properties of periodic bilayers and those constrained within nanodiscs, and figures of model parameter effects on shape profile, comparison of analytical solution and simulated profiles, radial lipid sorting in POPE–POPG nanodisc, and shape analysis methodology (PDF)
