# Supplementary material for: Capturing Lipid Nanodisc Shape and Properties Using a Continuum Elastic Theory
Source: J Chem Theory Comput. 2023 Feb 1;19(4):1360–9. doi: 10.1021/acs.jctc.2c01054 (PMC9979604; doi:10.1021/acs.jctc.2c01054)
Supplement: Supplementary file 1 — ct2c01054_si_001.pdf [file ct2c01054_si_001.pdf]

Supporting Information
Capturing Lipid Nanodisc Shape and Properties using Continuum Elastic Theory

Itay Schachter*,†,‡ and Daniel Harries‡

†*Institute of Organic Chemistry and Biochemistry of the Czech Academy of Sciences, Flemingovo nám. 542/2, CZ-16000 Prague 6, Czech Republic*

‡*Institute of Chemistry, the Fritz Haber Research Center, and the Harvey M. Kruger Center for Nanoscience & Nanotechnology, The Hebrew University, Jerusalem 9190401, Israel*

E-mail: daniel.harries@mail.huji.ac.il

S1. SUPPLEMENTARY TABLES

Table S1: Simulations details.

Environment	Lipid composition	T [K]	Number of lipids	Simulation time [ns]
Nanodisc MSP1E3D1[a]	DOPC	298	274	1000
Nanodisc MSP1[a]	DOPC	298	172	1000
Nanodisc MSP2N2	DOPC	298	676	125
Membrane[a]	DOPC	298	256	300
Nanodisc MSP2N2	POPE-POPG (75:25)	310	792	∼160
Membrane	POPE-POPG (75:25)	310	256	300
Nanodisc MSP1E3D1[a]	POPC	298	280	1000
Nanodisc MSP1[a]	POPC	298	176	1000
Nanodisc MSP2N2[a]	POPC	298	690	∼140
Membrane[a]	POPC	298	256	350
Nanodisc MSP1E3D1[a]	DLPC	303	298	1000
Nanodisc MSP1[a]	DLPC	303	186	1000
Membrane[a]	DLPC	303	256	300
Nanodisc MSP1E3D1[a]	DPPC	323	302	1000
Nanodisc MSP1[a]	DPPC	323	190	1000
Membrane[a]	DPPC	323	256	300
Nanodisc MSP1E3D1[a]	DEPC	298	286	1000
Nanodisc MSP1[a]	DEPC	298	180	1000
Membrane[a]	DEPC	298	256	500

[a]taken from Ref.[1]

Table S2: Physical properties of periodic lipid membranes.

Bilayer	$h_0{}^a$ [nm]	$K_C{}^b$ [pN nm]	$\kappa_t{}^c$ [pN nm^{-1}]	$K_a{}^d$ [pN nm^{-1}]
DEPC	1.905±0.003	60±1	40.4±0.2	123±8
DOPC	1.473±0.003	49.8±0.5	29.5±0.2	118±13
POPC	1.51±0.01	59.6±0.8	39.1±0.3	119±7
DPPC	1.528±0.001	84±1	42.3±0.2	103±7
DLPC	1.129±0.007	64.0±0.4	37.0±0.3	116±9
POPE-POPG (75:25)	1.602±0.002	100±1	55.5±0.3	95±15

[a]monolayer bulk thickness at carbonyl. [b]monolayer bending rigidity. [c]monolayer tilt modulus. [d]monolayer area compressibility modulus.

Table S3: Physical properties of lipid nanodiscs.

Nanodisc	Lipid composition	$r_{max}{}^a$ [nm]	$\overline{K_C}{}^b$ [pN nm]	$\overline{\kappa_t}{}^c$ [pN nm^{-1}]	δ^d [nm]	$\tilde{h}(r_{max})^e$ [nm]	$n_r(r_{max})^f$
MSP1	POPC	2.85	103±1	78±1	0.269	0.128	0.280
MSP1E3D1		3.45	95±1	63.9±0.6	0.193	0.116	0.255
MSP2N2		7.05	79±1	49.1±0.6	0.112	0.017	0.344
MSP1	DOPC	2.55	67±1	53.5±0.6	0.261	0.198	0.233
MSP1E3D1		3.75	59.7±0.6	41.6±0.3	0.156	0.115	0.232
MSP2N2		7.35	51±1	35.6±0,7	0.083	0.005	0.289
MSP1	DEPC	2.55	78±1	65±1	0.348	0.208	0.271
MSP1E3D1		3.45	84±1	65.2±0.8	0.259	0.161	0.261
MSP1	DPPC	2.55	183±3	124±3	0.307	0.218	0.208
MSP1E3D1		3.15	147±2	90±1	0.230	0.208	0.197
MSP1	DLPC	2.85	94±1	66.5±0.6	0.146	0.058	0.279
MSP1E3D1		3.15	84±1	52.2±0.5	0.089	0.104	0.145
MSP2N2	POPE-POPG (75:25)	7.65	90±2	64.5±0.6	0.088	-0.117	0.301

[a]radius of non-negligible MSP density. [b]averaged monolayer bending rigidity. [c]averaged bilayer tilt modulus. [d]excess average thickness. [e]excess thickness at the rim. [f]radial component of the lipid director near the rim.

S2. MOLECULAR DYNAMICS SIMULATIONS

Most simulations have been previously described in detail.[1] Additional simulations included 160 ns trajectory of MSP2N2 nanodisc comprised of POPE-POPG(75:25) lipids, a corresponding infinite bilayer following the same protocols given elsewhere[1] and 125 ns trajectory of MSP2N2 comprised of DOPC lipids, all were run using GROMACS 2021.4. GROMACS runs implemented a standard set of the input parameters also prescribed by CHARMM-GUI[2] which includes: van der Waals force switching turned on, cutoff 12 Å, switch distance 10 Å, PME for electrostatics, Parrinello-Rahman pressure coupling[3] (isotropic pressure coupling, tau_p 5 ps, compressibility 4.5e-5 bar^{-1}, ref_p 1 bar), Nose-Hoover thermostat for constant temperature control[4] (separated for membrane and solution), and integration timestep of 2 fs. See further details of simulations in Table S1 and in Ref.[1] All trajectories were post-processed before analysis.[1]

S3. SHAPE PROFILE FROM SIMULATIONS

Shape profiles were evaluated from simulations using the following procedure. Lipid positions were defined as the center of mass of the 2 carbonyl carbons of each lipid. The nanodisc lipid patch's center of mass was centered for each frame at the origin, and its midplane was aligned with the xy plane using principal component analysis over the lipids positions. The local instantaneous monolayer thickness h is defined as the distance between the lipids' position and the midplane, and the lipid director radial n_r and axial n_z components are defined as previously described in Refs.[1,5] Values of h, n_r and n_z are radially bin-averaged over 0.3 nm sized bins. Next, 9th degree polynomials functions for h, n_r and n_z were fitted to the bin-averaged values up to a distance of non-negligible protein count, defined as r_{max}. Due to radial symmetry, only even or odd terms in the polynomials were nonzero. For the large nanodiscs, this fit was performed only for 1.5 nm$< r < r_{max}$ as the thickness at the center did not fully converge over the relatively short simulation trajectories. Finally, in

fitting, the function $n_{r/z}(r)$ is replaced by the normalized $n_{r/z}(r)/(n_{r/z}(r)^2 + n_z(r)^2)^{1/2}$ so that $\boldsymbol{n}(r) = n_r(r)\boldsymbol{e}_r + n_z(r)\boldsymbol{e}_z$ is a unit vector.

S4. SHAPE ANALYSIS METHODOLOGY

S4.1. Model Numerical Solution

Due to rotational symmetry, in our model the surface of the confined monolayer is a surface of revolution of some curve $(r, h(r))$ around the z axis. The differential relation between the arc length and radial parametrizations is

$$ds = \sqrt{1 + (dh/dr)^2}dr \tag{S1}$$

The divergence of the lipid's director $\boldsymbol{n}(r)$ is

$$\nabla \cdot \boldsymbol{n} := \frac{\partial n_r}{\partial r} + \frac{\partial n_z}{\partial z} + \frac{n_r}{r} = \frac{dn_r}{ds}\frac{ds}{dr} + \frac{dn_z}{ds}\frac{ds}{dz} + \frac{n_r}{r} = \frac{d\boldsymbol{n}}{ds} \cdot \boldsymbol{\tau} + \frac{n_r}{r} \tag{S2}$$

The normal $\boldsymbol{N}(r)$ and tangent $\boldsymbol{\tau}(r)$ unit vector fields of this curve are defined as

$$\boldsymbol{N}(r) = \frac{-dh/dr}{\sqrt{1 + (dh/dr)^2}}\boldsymbol{e}_r + \frac{1}{\sqrt{1 + (dh/dr)^2}}\boldsymbol{e}_z = N_r\boldsymbol{e}_r + N_z\boldsymbol{e}_z \tag{S3}$$

$$\boldsymbol{\tau}(r) = N_z\boldsymbol{e}_r - N_r\boldsymbol{e}_z \tag{S4}$$

For clarity, the other relevant fields are the tilt $\boldsymbol{t}$ and relative area deviations α,

$$\boldsymbol{t} = \frac{\boldsymbol{n}}{\boldsymbol{n} \cdot \boldsymbol{N}} - \boldsymbol{N} \tag{S5}$$

$$\alpha \approx \frac{h - h_0}{h_0} - \frac{h_0}{2}\nabla \cdot \boldsymbol{n} \tag{S6}$$

and the area differential is

$$dS = 2\pi r ds \tag{S7}$$

Substituting Eqs.S1-S7 in Eq.1 of the main text results in a one-dimensional integral that is to be functionally minimized. In practice, $h(r)$ and $\theta(r) = \arcsin(n_r(r))$ are approximated as 9th order polynomials. Due to the cylindrical symmetry, odd polynomial terms are set to zero for the former and even terms for the latter. The interval $[0, r_0]$ is discretized into 300 equidistant points r_i, such that $dr < 0.03$ nm for $r_0 < 9$ nm, with half-indexed points denoted by $r_{i+0.5}$. The discretized form of elastic energy functional is

$$F_{\text{approx}} = \frac{1}{2} \sum_{i=0}^{298} dS_{i+0.5} \left[\left(K_{C,i+0.5} \left(\nabla \cdot \boldsymbol{n} \right)_{i+0.5} - J_s \right)^2 + \kappa_t \left(\boldsymbol{t}_{i+0.5} \right)^2 + K_a \left(\alpha_{i+0.5} \right)^2 \right] \tag{S8}$$

with the local value of the bending rigidity $K_{C,i} = K_C(r_i)$. The following expressions are calculated exactly:

$$\boldsymbol{n}_i = \sin(\theta(r_i))\boldsymbol{e}_r + \cos(\theta(r_i))\boldsymbol{e}_z \tag{S9}$$

$$h_i = h(r_i) \tag{S10}$$

$$\left(dh/dr \right)_i = h'(r_i) \tag{S11}$$

as well as the half-indexed terms. Using these expressions allows to evaluate $\boldsymbol{N}_{i+0.5}, \boldsymbol{\tau}_{i+0.5}, \boldsymbol{n}_{i+0.5}$ and $\boldsymbol{t}_{i+0.5}$. Other necessary half-indexed expressions for calculating Eq.S8 are given by,

$$ds_{i+0.5} = \sqrt{1 + \left(dh/dr \right)_{i+0.5}} dr \tag{S12}$$

$$dS_{i+0.5} = 2\pi r_{i+0.5} ds_{i+0.5} \tag{S13}$$

$$\left(\nabla \cdot \boldsymbol{n} \right)_{i+0.5} \approx \frac{n_{r,i+1} - n_{r,i}}{ds_{i+0.5}} \cdot \boldsymbol{\tau}_{i+0.5} + \frac{n_{r,i+0.5}}{r_{i+0.5}} \tag{S14}$$

$$\alpha_{i+0.5} \approx \frac{h_{i+0.5} - h_0}{h_0} - \frac{h_0}{2} \left(\nabla \cdot \boldsymbol{n} \right)_{i+0.5} \tag{S15}$$

Eq.S8 is minimized with respect to the coefficients of h, θ under the boundary condition

constraint $h(r_0), \theta(r_0)$, and the average excess thickness constraint

$$\delta = \int_0^{r_0} \frac{r}{r_0^2} \tilde{h}(r) dr \tag{S16}$$

which stems from the constant number of lipids within nanodiscs. This procedure is implemented via Python[6] and Scipy[7] using constrained minimization with the trust-constraint method,[8] where xtol and gtol are both set to 1e-9. This algorithm is repeated for the shape analysis under different values of K_C. The minimizing coefficients for each step are taken as the initial guess of the next, in order to ensure faster convergence. The initial guess is $\theta(r) = 0$ and h is set to the polynomial describing the thickness profile found in simulation.

S4.2. Fitting Procedure

To extract the bending modulus from the nanodisc shapes seen in simulations, we numerically fit the bending rigidity K_C so that the nanodisc shape minimizing Eq.S8 has the lowest RMSD from the one observed in simulations. Specifically, the optimization procedure minimizes the sum of least-square error of $N_r(r)$ and $n_r(r)$ up to a distance r_0 from the nanodisc center. In this procedure, the bulk values of κ_t, K_a and h_0 from the periodic membranes simulations are used. We set $r_0 = 1.9 < r_{max}$ nm for the medium and $r_0 = 0.7 r_{max}$ for the large nanodiscs. The boundary values $n_r(r_0), h(r_0)$ and δ (up to r_0) are taken to be their values in the simulations. For large nanodiscs, a thickness dependent K_C is assumed, by defining the trial function $A(h/h_0)^b + c$ pN nm, while requiring $1 \leq A, b$ and $0 \leq c$ to ensure that K_C is positive and correlated positively with h as well as to avoid local minimas solutions with constant K_C during the optimization. The optimization utilizes a combination of the basin hopping and Nelder-Mead algorithms[7,9] with $A = 30, b = 7, c = 50$ set as the initial guess. The resulting best-fit trial function is then applied to the thickness profile in simulations, which yields the predicted K_C profile up to r_{max}. For medium-sized nanodiscs, K_C is assumed spatially invariant, and determined by a bounded minimization fit using the

Brent method, for $K_C \in [10, 300]$[pN nm]. The spontaneous curvature J_s is set to zero for all but the large nanodisc composed of POPE-POPG for which we set it to -0.316[nm^{-1}], the experimental value of POPE at 310K.[10] because the other lipids have negligible spontaneous curvature.

S5. SUPPLEMENTARY FIGURES

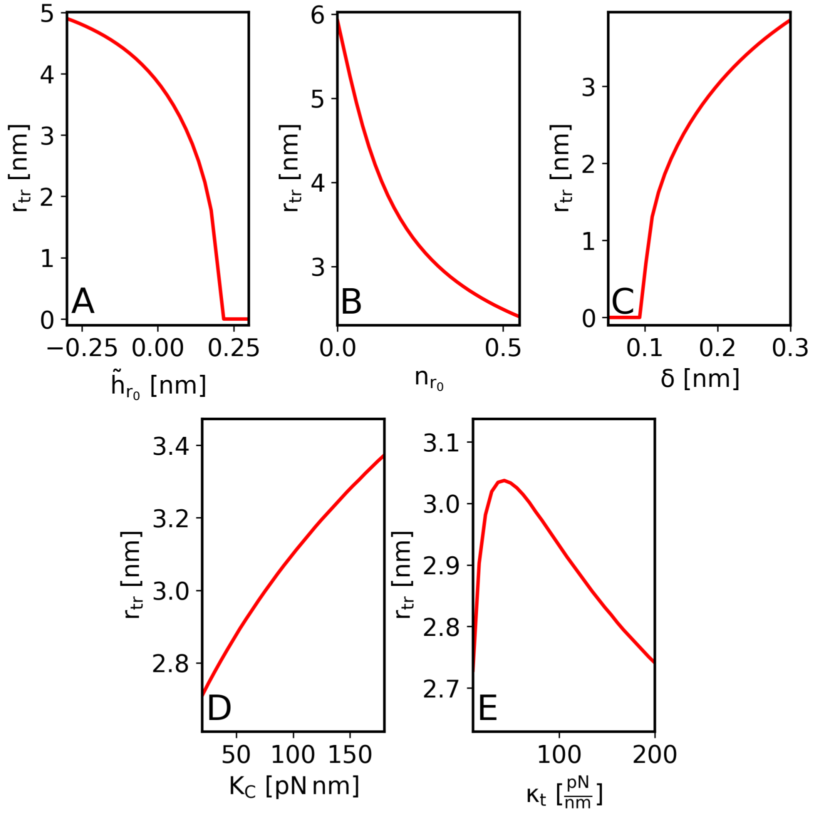

Figure S1: Influence of physical parameters on the radius r_{tr} where the thickness profile transitions from concave ($h'' < 0$) to convex ($h'' < 0$) at $r = 0$, as determined by the analytical solution. r_{tr} changes with (A) hydrophobic mismatch with the MSP, described by $\tilde{h}_{r_0}$, (B) lipid orientation at rim $n_r(r_0)$, (C) average excess thickness, δ, corresponding to lipid number density in nanodisc, (D) tilt modulus κ_t, and (E) bending modulus K_C. The variation in each parameter is performed around the following physical parameters, typical to lipids in a nanodisc: $K_C = 80$ pN nm, $\kappa_t = 60$ pN nm^{-1}, $K_a = 120$ pN nm^{-1}, $r_0 = 4$ nm, $n_r(r_0) = 0.3$, $\tilde{h}(r_0) = 0.1$ nm and $\delta = 0.2$ nm.

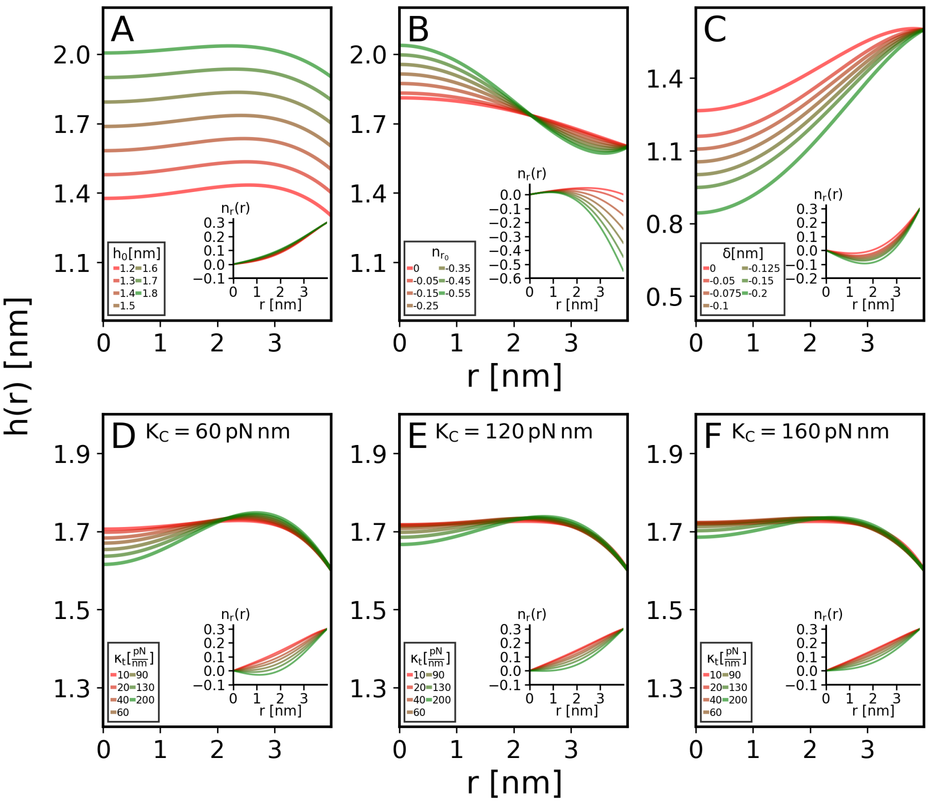

Figure S2: Influence of physical parameters on nanodisc lipid shape profile determined by the analytical solution. Each panel shows the influence of a single parameter on the radial lipid height (main panel) and lipid director projection (inset) profiles. Variations are shown for (A) bulk thickness h_0, (B) negative values of lipid orientation at rim, $n_r(r_0)$, (C) negative values of average excess thickness, δ, corresponding to lipid number density in nanodisc and (D-F) tilt modulus κ_t with different bending moduli: 60, 120, 160 pN nm. The variation is made around the following physical parameters, typical to lipids in a nanodisc, $K_C = 80$ pN nm, $\kappa_t = 60$ pN nm^{-1}, $K_a = 120$ pN nm^{-1}, $r_0 = 4$ nm, $n_r(r_0) = 0.3$, $\tilde{h}(r_0) = 0.1$ nm and $\delta = 0.2$ nm.

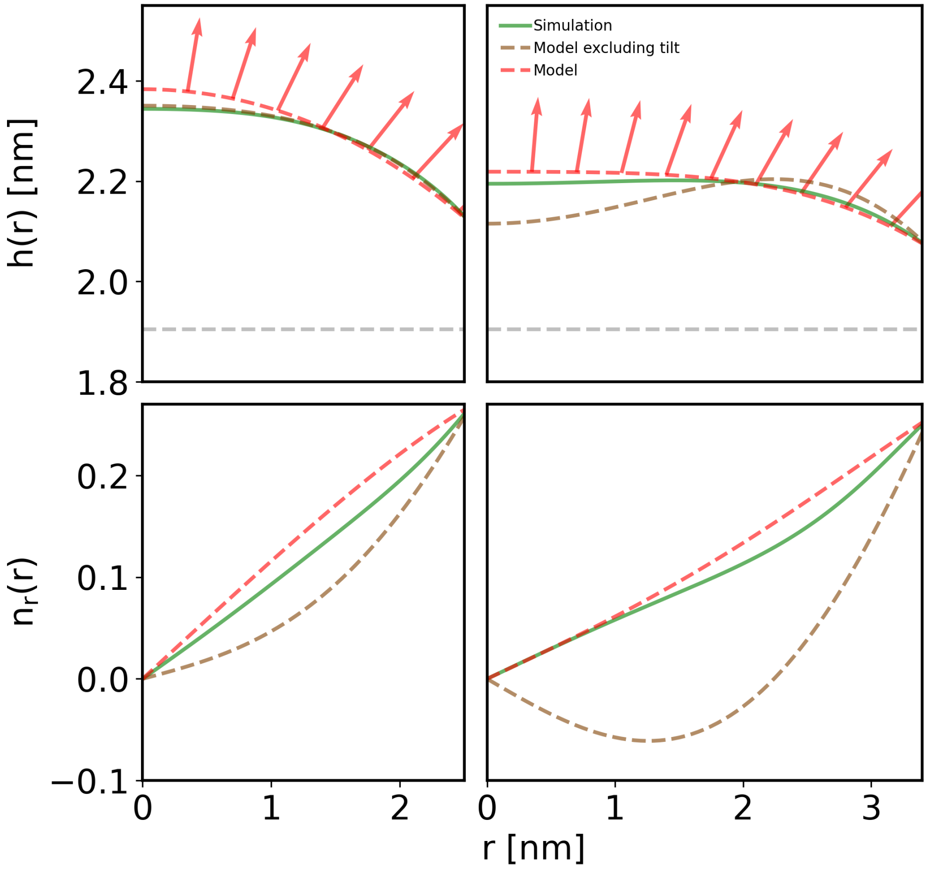

Figure S3: Comparison of nanodisc shape profiles found in simulation and calculated from continuum theory. The shape profile is described by the thickness (upper panels) and radial projection of the lipid director normal (lower panels) radial profiles. Shape profiles for DEPC lipid nanodiscs are shown for small (left column) and large (right column) nanodiscs. Profiles found through simulation (solid line) are compared with the analytical solutions (dashed lines) under matching model parameters, as detailed in the main text. Calculations are shown with or without including the tilt degree of freedom. The red arrows illustrate lipid director orientation in calculations which allow tilt, and these correspond to the dashed red curves in the lower panels. DEPC bulk thickness in simulations is shown for reference as a dashed gray horizontal line.

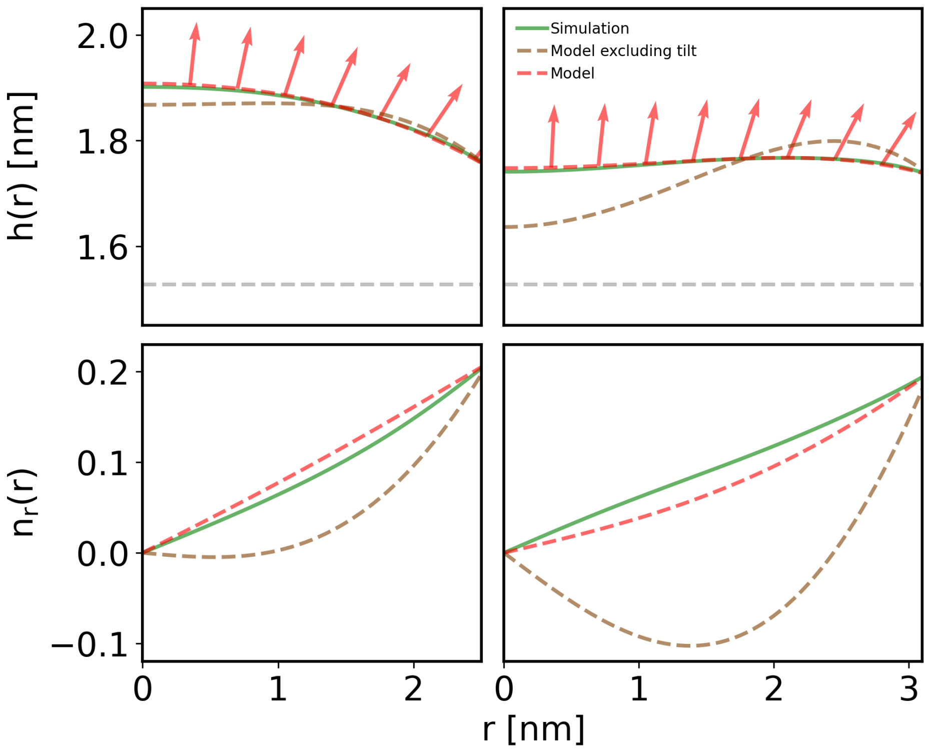

Figure S4: Comparison of nanodisc shape profiles found in simulation and calculated from continuum theory. The shape profile is described by the thickness (upper panels) and radial projection of the lipid director normal (lower panels) radial profiles. Shape profiles for DPPC lipid nanodiscs are shown for small (left column) and large (right column) nanodiscs. Profiles found through simulation (solid line) are compared with the analytical solutions (dashed lines) under matching model parameters, as detailed in the main text. Calculations are shown with or without including the tilt degree of freedom. The red arrows illustrate lipid director orientation in calculations which allow tilt, and these correspond to the dashed red curves in the lower panels. DPPC bulk thickness in simulations is shown for reference as a dashed gray horizontal line.

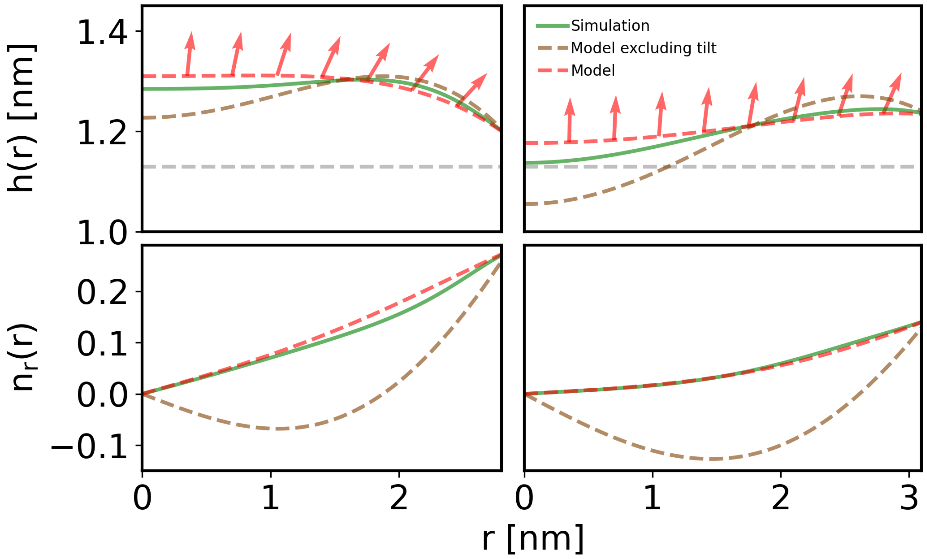

Figure S5: Comparison of nanodisc shape profiles found in simulation and calculated from continuum theory. The shape profile is described by the thickness (upper panels) and radial projection of the lipid director normal (lower panels) radial profiles. Shape profiles for DLPC lipid nanodiscs are shown for small (left column) and large (right column) nanodiscs. Profiles found through simulation (solid line) are compared with the analytical solutions (dashed lines) under matching model parameters, as detailed in the main text. Calculations are shown with or without including the tilt degree of freedom. The red arrows illustrate lipid director orientation in calculations which allow tilt, and these correspond to the dashed red curves in the lower panels. DLPC bulk thickness in simulations is shown for reference as a dashed gray horizontal line.

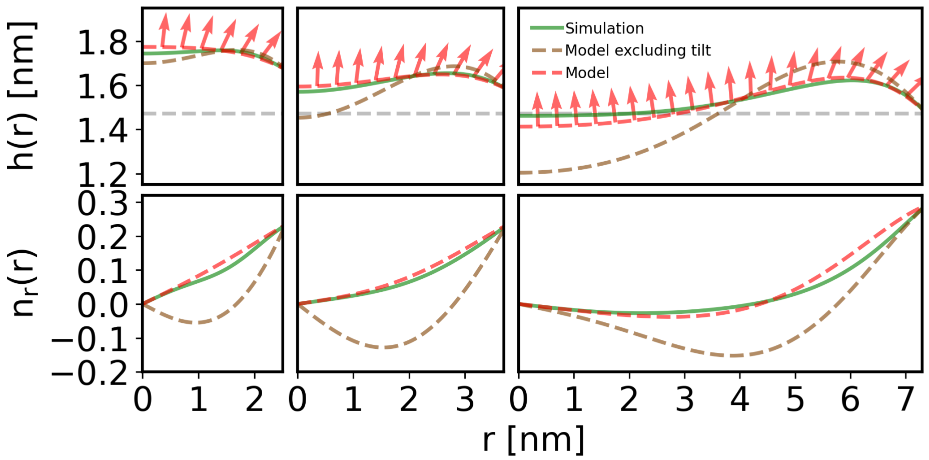

Figure S6: Comparison of nanodisc shape profiles found in simulation and calculated from continuum theory. The shape profile is described by the thickness (upper panels) and radial projection of the lipid director normal (lower panels) radial profiles. Shape profiles for DOPC lipid nanodiscs are shown for small (left column), medium (middle column) and large (right column) nanodiscs. Profiles found through simulation (solid line) are compared with the analytical solutions (dashed lines) under matching model parameters, as detailed in the main text. Calculations are shown with or without including the tilt degree of freedom. The red arrows illustrate lipid director orientation in calculations which allow tilt, and these correspond to the dashed red curves in the lower panels. DOPC bulk thickness in simulations is shown for reference as a dashed gray horizontal line.

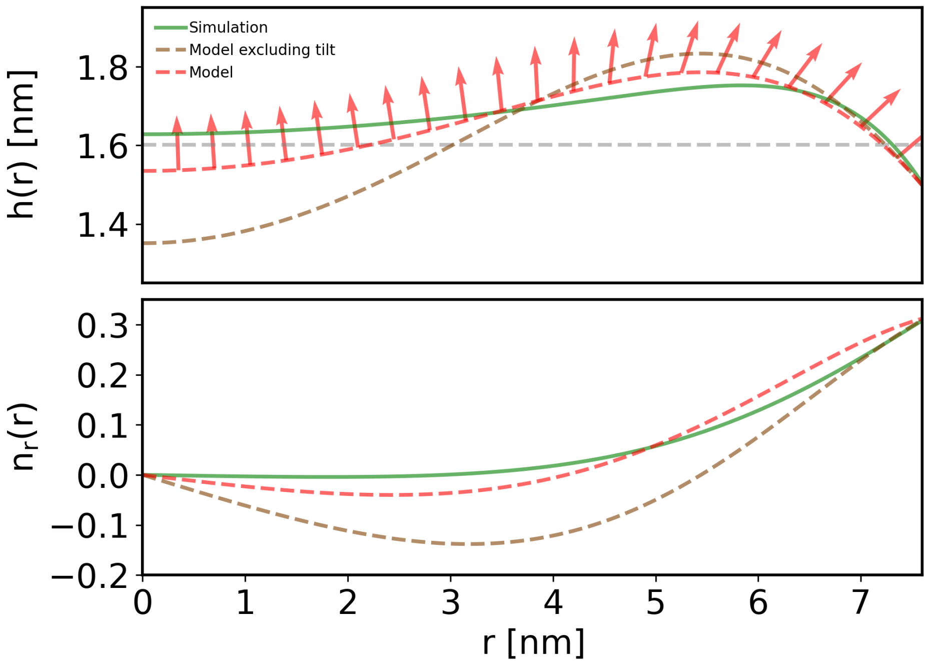

Figure S7: Comparison of nanodisc shape profiles found in simulation and calculated from continuum theory. The shape profile is described by the thickness (upper panel) and radial projection of the lipid director normal (lower panel) radial profiles. Shape profiles for POPE-POPG lipid large nanodisc are shown. The profile found through simulation (solid line) is compared with the analytical solutions (dashed lines) under matching model parameters, as detailed in the main text. Calculations are shown with or without including the tilt degree of freedom. The red arrows illustrate lipid director orientation in calculations which allow tilt, and these correspond to the dashed red curves in the lower panels. POPE-POPG bulk thickness in simulations is shown for reference as a dashed gray horizontal line.

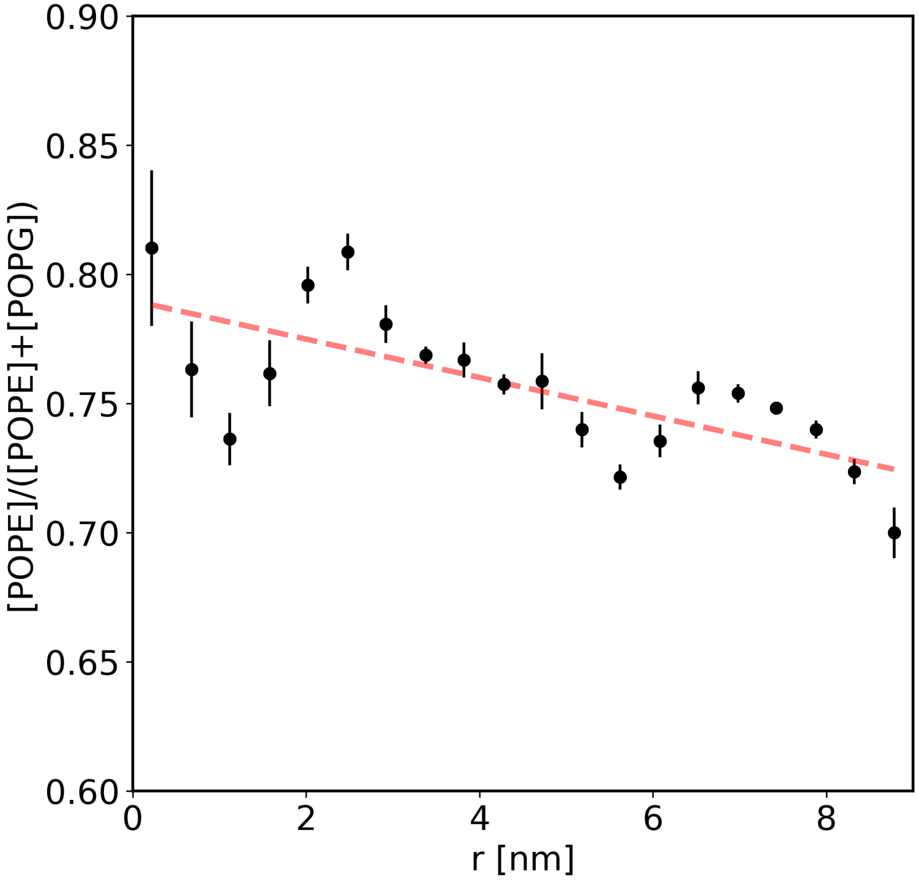

Figure S8: Radial lipid sorting in simulations of the large POPE-POPG nanodisc. The relative POPE lipid ratio is presented (scatter) with linear regression (dashed red line) as a visual guide. Errors were estimated via 10-chunk block averaging method.

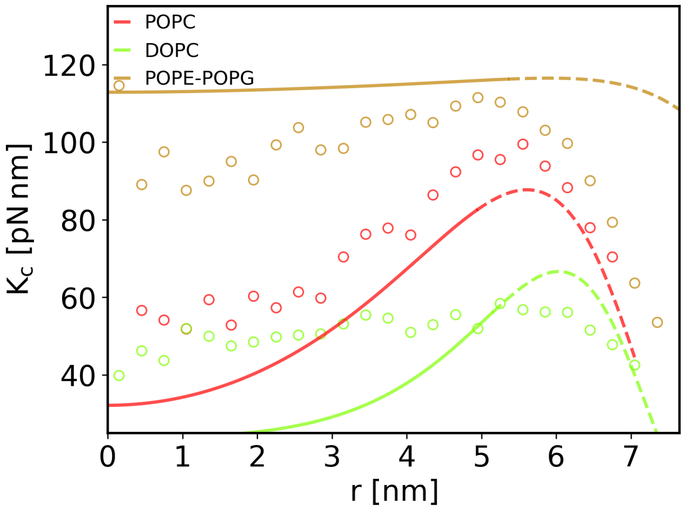

Figure S9: Bending rigidity radial profiles of large nanodiscs. The profiles were extracted from simulations using two different methods: real space fluctuations analysis (scatter) and through shape analysis performed on the shape profile defined at the third carbon up to a radius r_0 (solid lines) and extrapolated up to the radius of lipid-MSP contact r_{max} (dashed lines), as described in the main text.

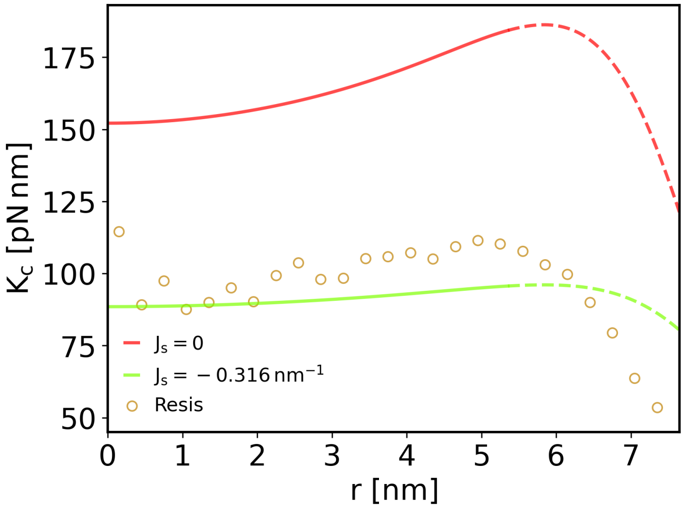

Figure S10: Bending rigidity radial profiles of large nanodiscs. The profiles were extracted from simulations through real space fluctuations analysis (scatter) and through shape analysis performed up to a radius r_0 (solid lines) and extrapolated up to the radius of lipid-MSP contact r_max (dashed lines) including or omitting the nonzero spontaneous curvatures J_s.

References

(1) Schachter, I.; Allolio, C.; Khelashvili, G.; Harries, D. Confinement in Nanodiscs Anisotropically Modifies Lipid Bilayer Elastic Properties. *J. Phys. Chem. B* **2020**, *124*, 7166–7175.

(2) Jo, S.; Kim, T.; Iyer, V. G.; Im, W. CHARMM-GUI: A Web-Based Graphical User Interface for CHARMM. *J. Comput. Chem.* **2008**, *29*, 1859–1865.

(3) Parrinello, M.; Rahman, A. Polymorphic Transitions in Single Crystals: A New Molecular Dynamics Method. *J. Appl. Phys* **1981**, *52*, 7182–7190.

(4) Evans, D. J.; Holian, B. L. The Nose–Hoover Thermostat. *J. Chem. Phys.* **1985**, *83*, 4069–4074.

(5) Allolio, C.; Haluts, A.; Harries, D. A Local Instantaneous Surface Method for Extracting Membrane Elastic Moduli from Simulation: Comparison with Other Strategies. *Chem. Phys.* **2018**, *514*, 31–43.

(6) Van Rossum, G.; Drake, F. L. *Python 3 Reference Manual*; CreateSpace: Scotts Valley, CA, 2009.

(7) Virtanen, P. et al. SciPy 1.0: Fundamental Algorithms for Scientific Computing in Python. *Nat. Methods* **2020**, *17*, 261–272.

(8) Conn, A. R.; Gould, N. I. M.; Toint, P. L. *Trust Region Methods*; Society for Industrial and Applied Mathematics, 2000; p 19.

(9) Avriel, M. *Nonlinear Programming: Analysis and Methods*; Prentice-Hall series in automatic computation; Prentice-Hall: Englewood Cliffs/N.J, 1977.

(10) Kollmitzer, B.; Heftberger, P.; Rappolt, M.; Pabst, G. Monolayer Spontaneous Curvature of Raft-Forming Membrane Lipids. *Soft Matter* **2013**, *9*, 10877–10884.
